# Perceived Stress in Obsessive–Compulsive Disorder is Related with Obsessive but Not Compulsive Symptoms

**DOI:** 10.3389/fpsyt.2013.00021

**Published:** 2013-04-02

**Authors:** P. Morgado, D. Freitas, J. M. Bessa, N. Sousa, João José Cerqueira

**Affiliations:** ^1^School of Health Sciences, Life and Health Sciences Research Institute, University of MinhoBraga, Portugal; ^2^ICVS-3Bs PT Government Associate LaboratoryBraga/Guimarães, Portugal; ^3^Centro Clínico Académico BragaBraga, Portugal

**Keywords:** obsessive–compulsive disorder, stress, cortisol, Y–BOCS, PSS-10

## Abstract

Obsessive–compulsive disorder (OCD) is achronic psychiatric disorder characterized by recurrent intrusive thoughts and/or repetitive compulsory behaviors. This psychiatric disorder is known to be stress responsive, as symptoms increase during periods of stress but also because stressful events may precede the onset of OCD. However, only a few and inconsistent reports have been published about the stress perception and the stress-response in these patients. Herein, we have characterized the correlations of OCD symptoms with basal serum cortisol levels and scores in a stress perceived questionnaire (PSS-10). The present data reveals that cortisol levels and the stress scores in the PSS-10 were significantly higher in OCD patients that in controls. Moreover, stress levels self-reported by patients using the PSS-10 correlated positively with OCD severity in the Yale–Brown Obsessive–Compulsive Scale (Y–BOCS). Interestingly, PSS-10 scores correlated with the obsessive component, but not with the compulsive component, of Y–BOCS. These results confirm that stress is relevant in the context of OCD, particularly for the obsessive symptomatology.

## Introduction

Obsessive–compulsive disorder (OCD) is a psychiatric disorder that affects 2–3% of population worldwide (Ruscio et al., 2010) and carries high levels of morbidity (Murray and Lopez, 1996; Hollander et al., 2010). It is characterized by obsessions (persistent, intrusive, and inappropriate thoughts, as well as impulses or images that cause anxiety) and compulsions (repetitive behaviors or thoughts performed in order to decrease the anxiety caused by the obsessions). Although genetic factors play an important role in the etiology of disease, several reports implicate environmental influences such as relevant life events and traumatic events in the onset of the disease (Zohar et al., 2007; Forray et al., 2010). Analyzing a group of 74 female OCD patients, Lochner et al. (2002) found them to have higher rates of childhood trauma than healthy controls. Interestingly, subsequent studies demonstrated that frequency, clinical pattern, and severity of OCD symptoms correlated not only with a history of one or more traumatic life events (Gershuny et al., 2003; Cromer et al., 2007; Real et al., 2011) but also with their intensity (Jordan et al., 1991).

Importantly, the stress-response and the activity of the hypothalamic-pituitary-adrenal (HPA) axis have been shown to be relevant in the context of several psychiatric disorders (Holsboer, 1983). This also holds true for OCD as it is known that stressful events may precede the onset of OCD (Toro et al., 1992) and that, in addition, OCD symptoms increase at times of stress (Findley et al., 2003). Nevertheless, it is also true that core symptoms of the disease, namely obsessions, cause significant distress, and as a consequence, may trigger physiological stress-related systems such as the HPA axis. However, the characterization of the activity of the HPA axis in OCD is still a matter of dispute, with some studies reporting normal levels of cortisol (Kuloğlu et al., 2007) and a normal dexamethasone suppression response (Monteiro et al., 1986; Jenike et al., 1987), while others observe high levels of cortisol (Gehris et al., 1990; Kluge et al., 2007) and non-suppression of cortisol during suppression tests (Cottraux et al., 1984; Catapano et al., 1990). Yet, the discrepancies extend beyond the measurement of cortisol levels; in fact, while one study showed increased corticotrophin releasing hormone (CRH) levels in the cerebrospinal fluid (Altemus et al., 1992) of OCD patients, which might be indicative of hyperactivation of stress-response systems, another found a decreased pituitary volume in OCD patients (Jung et al., 2009), which is suggestive of hypofunction of the adenohypophysis.

In light of such controversy, we thought of interest to further characterize the link between OCD and stress. In order to achieve this aim, we measured basal serum cortisol levels and assessed the perception of stress using a validated perceived stress scale 10 (PSS-10) in a group of OCD patients (without depression) and in a cohort of age- and sex-matched controls. In patients, such measurements were subsequently correlated with OCD symptoms, namely by discriminating the obsessive and compulsive components using the Yale–Brown Obsessive–Compulsive Scale (Y–BOCS).

## Materials and Methods

### Participants

The cohort under analysis comprised 18 patients with OCD and 18 healthy controls. Patients were admitted to the Psychiatric Department of Hospital de Braga as outpatients with a diagnosis of OCD. All patients were aged >18 years and able to communicate in Portuguese. Diagnosis was established by experienced psychiatrists with a semi-structured interview based on Diagnostic and Statistical Manual of Mental Disorders, Fourth Edition (DSM-IV)-TR and corroborated by a severity score of 7 or greater on the Y–BOCS. Exclusion criteria included: any other mental disorder revealed by the Mini-International Neuropsychiatric Interview (MINI Plus; except for OCD), any acute and/or chronic medical illness as assessed by a physical examination and routine laboratory examination, females who are pregnant or lactating and substance dependence within the previous 12 months. From 21 patients initially enrolled, 3 dropped out and thus only 18 were analyzed (2 patients were not included because they did not attend for blood collection and in the remainder case, blood sample was not collected properly). The three matching controls were also excluded from the analysis.

Healthy controls were carefully recruited to match OCD patients for age, sex, educational level, ethnical origin, and dominance. Exclusion criteria included previous history of neuropsychiatric disorder, any present mental disorder revealed by MINI Plus and use of any medication (excluding oral contraceptives).

All subjects provided written informed consent following a description of the procedures. The study protocol was approved by the Ethics Committee of the Hospital of Braga, Portugal. The study was performed in accordance with the Declaration of Helsinki.

### Instruments

#### Sociodemographic form

All subjects were evaluated by a semi-structured questionnaire form in order to characterize gender, age, marital status, educational level, professional status, ethnical origin, and previous medical history of the cohorts. OCD patients were also evaluated in terms of duration of illness, type of obsessions, and compulsions and medication taken. Table 1 summarizes the characteristics of patients and healthy controls.

**Table 1:** **Sociodemographic and clinical characteristics of patients with obsessive–compulsive disorder and healthy comparison subjects**.

Characteristics	Subjects with OCD (*n* = 18)	Healthy comparison subjects (*n* = 18)	Statistics
Age, years [mean ± SD (range)]	27.33 ± 6.11 (21–38)	26.28 ± 5.21 (20–38)	*P* = 0.691
Male/female	12/6	12/6	
Education, years [mean ± SD (range)]	13.22 ± 1.99 (12–18)	14.06 ± 3.37 (12–18)	*P* = 0.346
Body mass Index [mean ± SD (range)]	23.70 ± 4.18 (17–31)	22.78 ± 2.18 (19–29)	*P* = 1.000
Age of onset [mean ± SD (range)]	21.61 ± 7.05 (9–35)		
Duration of illness [mean ± SD (range)]	5.72 ± 6.70 (0–21)		
Y–BOCS (total score)	25.61 ± 5.90 (12–30)		
Y–BOCS (obsession score)	13.50 ± 3.17 (7–20)		
Y–BOCS (compulsion score)	12.11 ± 3.27 (5–17)		
HDRS (global score)	3.83 ± 2.53 (0–7)		
HARS (global score)	4.33 ± 3.20 (0–16)		
Medication	Only SSRI – 14 (77.8%); SSRI with TCA – 4 (22.2%)		

*OCD, obsessive–compulsive disorder; Y–BOCS, Yale–Brown obsessive–compulsive scale; HDRS, Hamilton depression rating scale; SSRI, serotonin selective reuptake inhibitors; TCA, tricyclic antidepressant; HARS, Hamilton anxiety rating scale*.

#### Mini-international neuropsychiatric interview

Patients were assessed with MINI Plus, a short structured diagnostic interview (Sheehan et al., 1998), design to screen for neuropsychiatric diagnosis according to the DSM-IV.

#### Yale–Brown obsessive–compulsive scale

Yale–Brown Obsessive–Compulsive Scale was used to assess the severity of OCD and to discriminate the symptoms sub-components of the disorder. The Y–BOCS is composed of 10-items, half related with obsessions and the other half related with compulsions. Each item is assessed by a clinician and rated on a five-point likert-type scale from 0 to 4 (Goodman et al., 1989).

#### Perceived stress scale 10

The Portuguese version of the 10-items Perceived Stress Scale, filled-out on the same day of blood collection, was used to assess perception of stress (Cohen et al., 1983). Items were classified on a five-point likert-type scale from 0 (never) to 4 (very often), and refer to the last month. The higher the total score, the greater the intensity of stress perceived by the subject.

#### Hamilton depression rating scale

This 17-items scale is used to rate the severity of depression (Hamilton, 1960). Scores higher than 25 indicate severe depression while scores below 7 indicate no depression.

#### Hamilton anxiety rating scale

Fourteen-items scale used to evaluate severity of anxiety (Hamilton, 1959). Each item is scored on a scale of 0 (not present) to 4 (severe). Scores higher than 25 indicate moderate/severe anxiety while scores below 17 indicate mild symptoms.

#### Blood sampling

Venous blood samples from left forearm vein were collected into 5 mL tubes containing potassium EDTA, between 1:00 and 4:00 p.m. Precise instructions about sleep and alimentation were given to volunteers that should not have any food neither drink except water in the 6-h prior the blood collection. Plasma was separated by centrifugation and stored at −70°C. Serum cortisol was determined by standard radioimmuno assays.

### Statistical analysis

Data was analyzed using SPSS (version 19.0; IBM). Demographics, clinical measures, psychometric scales, and laboratory values were reported using descriptive statistics (frequencies, means, and standard deviation). Group comparisons were carried out by non-parametric Mann–Whitney *U* test to compare means. For correlation evaluations, the Pearson correlation test was used. Differences were considered to be significant if *p* < 0.05.

## Results

No significant differences in age, gender, education, or body mass index were found between the OCD group and controls (Table 1). Three OCD patients and four healthy controls were taking oral contraceptives, but no major differences/trends were observed between those on and without them.

The mean OCD severity was 25.61 as measured by Y–BOCS; there was no significant difference between the obsessive and the compulsive sub-scores in OCD patients (Table 1). The mean depression score [measured by Hamilton Depression Rating Scale (HDRS)] was 3.83; importantly, no subject displayed values above 7. The mean anxiety score [measured by Hamilton Anxiety Rating Scale (HARS)] was 4.33. The mean age of onset of the disease was 21.61 years and mean duration of illness was 5.72 years. All patients were medicated at the time of study, 77.8% with fluvoxamine alone (200–300 mg/day) and 22.2% with fluvoxamine and clomipramine (200–300 and 75–150 mg/day, respectively). The clinical characteristics of patients are summarized in Table 1.

**Figure 1:**
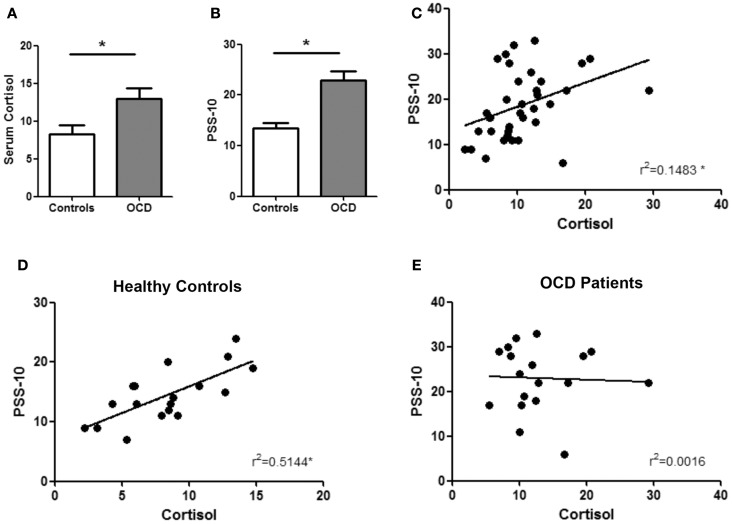
**Stress response in OCD patients and controls**. OCD patients shown high basal levels of serum cortisol (12.98 ± 5.77 mg/dL) when compared with healthy controls (8.28 ± 3.60 mg/dL) **(A)**. In accordance, OCD patients score higher in the perceived stress scale (PSS-10) questionnaire **(B)**. Importantly, these two measurements were positively correlated **(C)**. Looking at each group separately, cortisol and PSS-10 score correlate in healthy controls **(D)** but not in OCD patients **(E)** *p < 0.05.

The basal serum concentration of cortisol was significantly higher in OCD patients than in healthy controls (*P* = 0.011) (Figure 1A). Stress perception, as assessed by PSS-10, was significantly higher in OCD patients than in control subjects (*P* ≤ 0.001) (Figure 1B). Importantly, we found a positive correlation between these two measurements of response to stress in our entire population (*r* = 0.385, *P* = 0.020) (Figure 1C) that was only replicated in control subjects (*r* = 0.717, *P* = 0.001) (Figure 1D) but not in OCD patients (*r* = 0.040, *P* = 0.874) (Figure 1E).

**Figure 2:**
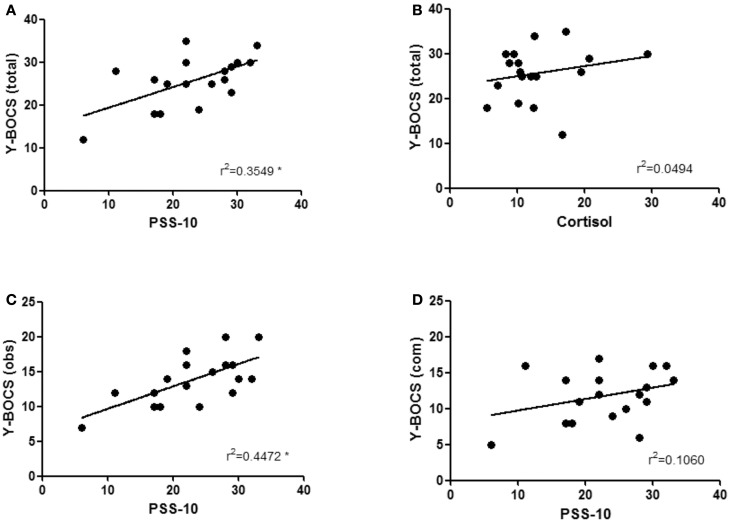
**Obsessive–compulsive disorder severity and stress-related measures**. OCD severity, as measured by Y–BOCS, positively correlate with PSS-10 score **(A)**, but not with serum cortisol levels **(B)**. Looking at each dimension of the Y–BOCS scale separately, there is a positive correlation between perceived stress and the obsessive score **(C)** but not with the compulsive score **(D)** *p < 0.05.

Stress self-reported by patients using PSS-10 positively correlated with OCD severity as assessed by Y–BOCS (*r* = 0.596, *P* = 0.009) (Figure 2A). Interestingly, no significant correlation was found between cortisol levels and OCD severity as assessed by Y–BOCS (*r* = 0.222, *P* = 0.376) (Figure 2B). Importantly, the score on the PSS-10 correlated significantly with obsessive component of Y–BOCS (*r* = 0.669, *P* = 0.002) (Figure 2C), but not with the compulsive sub-score (*r* = 0.326, *P* = 0.187) (Figure 2D).

## Discussion

In this study, we show that OCD patients report significantly higher levels of perceived stress than healthy controls, and that these are accompanied with higher serum cortisol levels. These findings support the hypothesis that dysregulated stress-response mechanisms are of relevance to this disorder. In this regard, it is important to note that, in our study, self-reported perceived stress levels also correlated positively with global severity of OCD, further strengthening the relevance of our data. Interestingly, these results are in line with a study by Jordan et al. (1991) in which previous traumatic events correlated with the intensity of OCD symptoms. Our data also shows that perceived stress is significantly correlated with the intensity of obsessive symptoms, but not with the intensity of compulsions. Indeed, while obsessions are highly stressful and anxiogenic ideas, compulsive actions are usually perceived as stress relieving. Of note, this finding is in accordance with previous studies that reports that OCD patients suffer significantly more stress by daily events (Coles et al., 2005) and that there is an important relationship between distress tolerance and obsessions (Cougle et al., 2011).

Although self-reported stress was highly correlated with illness severity and obsessive component of Y–BOCS, this study fails to demonstrate correlations between cortisol levels and OCD global severity or each OCD specific component. These can be explained by the recruitment of alternative systems of stress-response but also by the dynamic balance between obsessions and compulsions. High levels of cortisol were reported in previous studies (Gehris et al., 1990; Kluge et al., 2007), even though one study has observed that cortisol elevation was only related with co-morbid depressive symptoms (Kuloğlu et al., 2007). Despite these inconsistent reports, several findings such as non-suppression on dexamethasone test (Cottraux et al., 1984; Catapano et al., 1990), elevation of nocturnal ACTH (Kluge et al., 2007), and reduced pituitary volumes in non-treated OCD patients (Jung et al., 2009) support the altered functioning of HPA axis. Additionally, results from a study that analyzes therapeutic effects and hormonal changes induced by intravenous citalopram treatment suggest that the drug effects are dependent on cortisol response to SSRI (Corregiari et al., 2007) which can be related with cortisol modulation of 5-HT1A post-synaptic activity (Karten et al., 1999; Bijak et al., 2001). Interestingly, we report significant elevations of cortisol levels in a group of OCD patients that are receiving treatment for more than 6 weeks but remain with significant symptoms of disease.

The stress-related findings pointed out by this work are not specific of OCD and can be found in other psychiatric disorders such as depression. However, dysfunction in orbitofronto-striatal circuits has been the most common finding in the pathophysiology of OCD and previous animal and human studies have shown that these circuits are highly sensitive and can be disrupted by chronic stress, inducing a shift in observed decision-making behaviors through habits (Dias-Ferreira et al., 2009; Soares et al., 2012) and impairing ability of associate environmental cues to goal-directed behaviors (Morgado et al., 2012). Altogether, these observations support a possible role for chronic stress in the etiology of OCD.

By using a significantly homogeneous group of patients that did not display any comorbidity, we eliminate some frequent biases observed in other studies. However, this study has some methodological limitations that should be taken into account: first, we included only medicated patients with OCD, which might bias results; second, this study has a cross-sectional design; and finally, the size of the sample is relatively small.

In summary, this work highlights the dysfunction of stress perception and stress-response systems in the OCD. However, more studies are necessary to clarify whether these findings are implicated in the onset of the symptomatology or are a mere consequence of the symptoms.

## Conflict of Interest Statement

The authors declare that the research was conducted in the absence of any commercial or financial relationships that could be construed as a potential conflict of interest.
